# Official Business

**Published:** 1913-01

**Authors:** 


### Official Business.

Missionary—"Why do you look at me so intensely?"

Cannibal—"I am the food inspector."—Buffalo Commercial.

